# No Effects of Acute Psychosocial Stress on Working Memory in Older People With Type 2 Diabetes

**DOI:** 10.3389/fpsyg.2020.596584

**Published:** 2021-01-07

**Authors:** Lorena Vallejo, Mariola Zapater-Fajarí, Teresa Montoliu, Sara Puig-Perez, Juan Nacher, Vanesa Hidalgo, Alicia Salvador

**Affiliations:** ^1^Laboratory of Social Cognitive Neuroscience, Department of Psychobiology and University Institute for Research in Psychology of Human Resources, Organizational Development and Quality of Work Life (IDOCAL), University of Valencia, Valencia, Spain; ^2^Department of Health Sciences, Valencian International University, Valencia, Spain; ^3^Valencian (VLC) Campus Research Microcluster “Technologies of Information and Control Applied to the Pathophysiology and Treatment of Diabetes,” University of Valencia, Valencia, Spain; ^4^Centro de Investigación Biomédica en Red de Salud Mental: Spanish National Network of Research in Mental Health, Madrid, Spain; ^5^II Aragón, Department of Psychology and Sociology, Area of Psychobiology, University of Zaragoza, Teruel, Spain

**Keywords:** psychosocial stress, working memory, type 2 diabetes, older adults, cortisol, alpha-amylase

## Abstract

Type 2 diabetes (T2D) has been considered a public health threat due to its growing prevalence, particularly in the older population. It is important to know the effects of psychosocial stress and its potential consequences for some basic cognitive processes that are important in daily life. Currently, there is very little information about how people with T2D face acute psychosocial stressors, and even less about how their response affects working memory (WM), which is essential for their functionality and independence. Our aim was to characterize the response to an acute laboratory psychosocial stressor and its effects on WM in older people with T2D. Fifty participants with T2D from 52 to 77 years old were randomly assigned to a stress (12 men and 12 women) or control (12 men and 14 women) condition. Mood and physiological (cortisol, C, and salivary alpha-amylase, sAA) responses to tasks were measured. In addition, participants completed a WM test before and after the stress or control task. Our results showed that the TSST elicited higher negative affect and greater C and sAA responses than the control task. No significant differences in WM were observed depending on the exposure to stress or the control task. Finally, participants who showed higher C and sAA responses to the stressor had lower WM performance. Our results indicate that medically treated older adults with T2D show clear, typical mood and physiological responses to an acute psychosocial stressor. Finally, the lack of acute psychosocial stress effects on WM suggests that it could be related to aging and not to this disease, at least when T2D is adequately treated.

## Introduction

Type 2 diabetes (T2D) has been considered a public health threat (Rucker et al., 2012) because of its growing prevalence (Exalto et al., 2013), particularly in the older population (Rucker et al., 2012). Disturbances in blood glucose (BG) homeostasis in these individuals can lead to transient or permanent alterations, such as decreased psychomotor speed and memory deficits (Yeung et al., 2009). In addition, learning also seems to be very sensitive to glycemic variations (Ryan and Geckle, 2000) and poorer metabolic control (Ryan et al., 2016), which are associated with the development of cognitive complications (Ryan et al., 2006) and a faster decline on memory measures compared to normo-glycemic subjects (Tuligenga et al., 2014). Furthermore, reduced frontal lobe/executive function has been found in the T2D population, including in certain memory domains such as working memory (WM) (Kim, 2019). Prefrontal cortex also contributes to other cognitive functions, such as reasoning, planning, and decision-making (Funahashi, 2015, 2017), which are essential for well-being and quality of life. Hence, the impact of T2D on these cognitive functions is a crucial topic that should be addressed.

In this sense, Ryan et al. (2006) reported that subjects with poor glycemic control had difficulty performing the WM tasks. Furthermore, WM impairments associated with episodes of both hypoglycemia (Aung et al., 2012) and hyperglycemia (Cerasuolo and Izzo, 2017) have been found in diagnosed T2D patients. However, Arvanitakis et al. (2006) concluded that T2D was not associated with worse WM performance.

There is a large body of evidence about the relationship between stress and T2D. In fact, chronic stress seems to play an important role in the etiology (Surwit et al., 1992) and development of T2D (Agardh et al., 2003; Heraclides et al., 2009; Eriksson et al., 2013; Hackett and Steptoe, 2016). In this line, Mommersteeg et al. (2012) showed that high psychological distress was a risk factor for the development of diabetes in an 18-year follow-up study. Furthermore, a prospective 35-year follow-up study reported that high perceived stress in adulthood increased the risk of T2D (Novak et al., 2012). It has been well established that the exposure to stress is associated with the activation of both the sympathetic nervous system (SNS) and the hypothalamic-pituitary-adrenal (HPA) axis. Consequently, large amounts of alpha-amylase (an indirect marker of SNS) and cortisol (C), the main human glucocorticoid, are secreted. The chronic activation of these two systems produces an increase in BG levels, contributing to T2D development (Boaz et al., 2013; Lundqvist et al., 2019).

Regarding acute stress, higher glucose and C levels were reported in T2D patients after exposure to a psychosocial stressor (i.e., Trier Social Stress Test, TSST), in comparison with a control session (Faulenbach et al., 2011). However, significant C decreases immediately after a mental stressor have also been found and interpreted as a disruption in stress-related biological systems (Steptoe et al., 2014; Carvalho et al., 2015). To the best of our knowledge, no previous study has investigated the salivary alpha-amylase (sAA) response to an acute stress in T2D patients. Only one study has analyzed the relationships between sAA levels and perceived stress in T2D patients (Siddiqui et al., 2015), and it reported a positive relationship between them.

It has been well established that acute stress affects memory processes (Lindau et al., 2016; Hidalgo et al., 2019), given that there is an overlap of the brain structures involved in the stress response and memory processes. Specifically, the prefrontal cortex and the hippocampus, brain areas with a high density of glucocorticoid receptors, are involved in WM (Galloway et al., 2008; Kumar et al., 2016). It is important to note that most of the studies investigating stress-induced cortisol effects on WM have been carried out in healthy young people (for a review see Hidalgo et al., 2019), with only one in healthy older people (Pulopulos et al., 2015). In this study, the authors failed to find acute stress effects on WM performance, even though participants showed C and sAA responses to the TSST.

In addition, there is evidence of an association between cognitive performance in T2D and hippocampal integrity (Jones et al., 2014). This structure is especially sensitive to the effects of hypoglycemia (Marks and Rose, 1965) and higher BG levels, leading to hippocampus and amygdala atrophy (Cherbuin et al., 2012). Hippocampal atrophy was found in individuals with T2D, which could correlate with impairments in immediate memory (Gold et al., 2007). However, no previous studies have investigated the acute effects of stress on WM in T2D older people in spite of, as noted above, T2D patients show deficits in WM (for reviews see: Palta et al., 2014; Pelimanni and Jehkonen, 2018; Kim, 2019).

Hence, the aim of the present study was to assess the psychobiological (i.e., mood, sAA, and C) response to an acute stressor (i.e., TSST) and its effect on WM performance in diagnosed and medically treated older people with T2D. We expected that participants in the stress condition would show increases in negative mood and decreases in positive mood (Almela et al., 2011; Hidalgo et al., 2020) and higher C and sAA levels (Almela et al., 2011; Pulopulos et al., 2015) than participants in the control condition. In spite of the lack of significant findings in healthy older people (Pulopulos et al., 2015), based on worse WM performance in people with T2D (Gorniak et al., 2018), we expected that acute stress would impair WM performance.

## Materials and Methods

### Participants

The final sample was composed of 50 participants from 52 to 77 years old, 24 men and 26 women, with a medical diagnosis of T2D. They were randomly assigned to two conditions in a counter-balanced way: 24 to the stress condition (12 men and 12 women) and 26 to the control condition (12 men and 14 women). No gender differences were found between the two conditions (X^2^ = 0.074, *p* = 0.786) (see Table 1 for sample characteristics).

**TABLE 1:** Descriptive statistics (mean ± SEM) for total sample and both experimental conditions.

	Total sample (N = 50)	Stress Condition (N = 24)	Control Condition (N = 26)	*t, X^2^*
Age (years)	65.64 (0.724)	66.54 (0.936)	64.81 (1.083)	*t* = 1.202, *p* = 0.235
BMI (kg/m^2^)	28.77 (0.725)	28.411 (0.993)	29.094 (1.059)	*t* = −0.467, *p* = 0.643
SES	5.233 (0.216)	5.348 (0.324)	5.114 (0.290)	*t* = 0.536, *p* = 0.594
Time diagnosed (years)	11.02 (1.114)	10.75 (1.592)	11.33 (1.583)	*t* = −0.259, *p* = 0.797
Educational level (%)				*X*^2^ = 3.240, *p* = 0.356
No studies	6.1	0	11.5	
Basic studies	34.7	39.1	30.8	
High school	28.6	26.1	30.8	
College or higher	30.6	34.8	26.9	
Marital status (%)				*X*^2^ = 3.265, *p* = 0.353
Single	18.4	21.8	15.4	
Married	65.3	65.2	65.4	
Divorced	10.2	13	7.7	
Widowed	6.1	0	11.5	

*%, percentages; BMI, body mass index; SES, subjective socioeconomic status-scale (Adler et al., 2000; from 1: lowest to 10: highest level).*

Participants were recruited from hospitals and medical clinics and referred by their doctors; all of them were being medically treated and controlled during the period of the research. All volunteers were interviewed by telephone in order to find out if they met the exclusion criteria, which were: smoking more than 10 cigarettes per day, abuse of alcohol or other drugs of abuse, presence of severe cardiovascular disorder, psychiatric or neurological disorder, visual or hearing impairments, having been under general anesthesia in the past year, the presence of a stressful life event in the past year, and consuming drugs related to cognitive or emotional functions or psychotropic substances. As T2D patients, they were medicated as follows: 54.5% oral antidiabetic, 34.1% oral and injectable, and 11.4% only injectable. Additionally, volunteers who used other medications that could influence hormonal levels (glucocorticoids, beta-blockers, antidepressants, asthma medication, or thyroid therapies) were excluded from the study.

All participants received verbal and written information about the study and signed an informed consent form. The study was carried out in accordance with the Declaration of Helsinki, and the Ethics Research Committee of the University approved the protocol.

### Procedure

Participants attended an individual session held between 16:00 and 18:00 in the Laboratory of Social Cognitive Neuroscience at the University of Valencia. Before the session, the experimenter first checked whether each participant had followed the instructions prior to participation: refrain from heavy physical activity from the evening before the session, sleep as long as usual, and not consume alcohol since the night before the session. Additionally, participants were instructed to drink only water and not eat, smoke, take any stimulants (e.g., coffee, cola, tea, chocolate), or brush their teeth at least 2 h prior to the session.

#### Stress Condition

To produce stress, we employed the TSST (Kirschbaum et al., 1993). This task consisted of 5 min of free speech (simulated job interview) and 5 min of an arithmetic task. Participants stood in front of a committee with a video camera and a microphone clearly visible. Before the TSST, participants completed several items to assess their perceived self-efficacy and the PANAS (pre-task PANAS) to obtain the baseline measure for mood. In addition, WM was evaluated with Letters and Number Sequencing (pre-task LNS). Immediately after the TSST, participants filled in some items about the task (Situational Appraisal), the PANAS (post-task PANAS), and the LNS (post-task LNS).

#### Control Condition

This condition was similar to the stress condition in terms of mental workload and global physical activity, but it lacked the main components that could provoke stress, such as evaluative threat and uncontrollability (Dickerson and Kemeny, 2004). The control task consisted of 5 min of free speech about a recent non-emotional experience, followed by an arithmetic task consisting of 5 min of counting by 5 aloud. This control task has been used in previous studies (Pulopulos et al., 2013, 2019; Puig-Perez et al., 2015).

**FIGURE 1:**
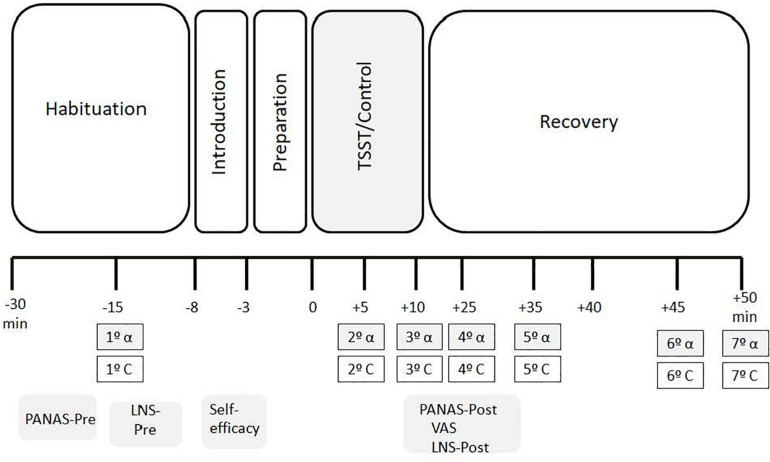
Timeline of the TSST (S) and control (C) conditions. Salivary cortisol samples = 1^o̱^ C, 2^o̱^ 3^o̱^ C, 4^o̱^ C, 5^o̱^ C, 6^o̱^ C, 7^o̱^ C Salivary alpha-amylase samples = 1^o̱^α, 2^o̱^α 3^o̱^α, 4^o̱^α, 5^o̱^α, 6^o̱^α, 7^o̱^α. Time of collection of saliva samples (−15, +5, +10, +25, +35j +45, +50). TSST, trier social stress test; PANAS, positive and negative affect; LNS, letter-number sequencing; VAS, situational appraisal.

During each session, we collected seven saliva samples to measure sAA and C levels. Specifically, the first saliva sample was collected prior to the stress/control task at habituation (−15 min pre-stress). The rest of the saliva samples were taken during the stress/control task (+5 min), after the TSST/Control task (+10 min post-stress), three at 10 min periods after the task (+25, +35, and +45 min), and finally at +50 min post-stress. In addition, two capillary blood samples were taken at habituation (−20 min) and in the recovery period (+55 min) to measure glucose concentrations. Both conditions followed the same time schedule for sample collection, phase durations, and questionnaires administered, and they only differed on the task (Stress vs. Control) (see Figure 1).

### Psychological Assessment

#### Self-Efficacy

Participants’ self-efficacy, understood as beliefs about one’s capacity to organize and carry out actions (Bandura, 1997), was evaluated with three items (van der Meij et al., 2010; Villada et al., 2017). These items were formulated to measure capacity (“How capable are you of successfully performing the task?”), self- confidence (“How confident are you that you will successfully perform the task?”), and importance of successfully performing the task (“How important is it to you to perform this task successfully?”). Participants had to respond on a Likert scale ranging from “1 = not at all” to “100 = totally.”

#### Situational Appraisal

Several items were used to measure the participants’ situational appraisal (Gonzalez-Bono et al., 2002), focusing on the following aspects: frustration, motivation, amount of perceived stress, difficulty of the task, assessment of their own performance, expected outcome of the evaluation, and perception of the effort required (e.g., How much effort did the task require?). Each item was rated on a 5-point Likert scale (not at all = 1 to extremely = 5).

#### Mood

The Spanish version (Sandín et al., 1999) of the Positive and Negative Affect Scale (PANAS) (Watson et al., 1988) was employed. This 20-item questionnaire assesses mood in two dimensions: positive affect (PA: interested, excited, strong, enthusiastic, etc.) and negative affect (NA: distressed, upset, guilty, scared, etc.), with 10 items measuring each state. The participants were asked to complete the questionnaire based on how they felt at that particular moment. They responded using a 5-point Likert scale ranging from 1 (not at all) to 5 (extremely). In the current study, Cronbach alpha coefficients were 0.91 and 0.87 for PA and NA, respectively.

### Working Memory Task

The Letter–Number Sequencing (LNS) task from the Wechsler Memory Scale III (Wechsler, 1997) was used to assess WM performance. This test requires participants to listen to sequences of alternating digits (ranging from 0 to 9) and letters (from A to Z) of increasing lengths. Then, they have to repeat the digits and letters from each sequence, beginning with the digits in numerical order and then the letters in alphabetical order. The length of the sequences increased from two to eight items, and participants were allowed three attempts to solve each sequence length. One point was assigned for each correct attempt, and the task ended when the participant had failed the three attempts for the same sequence length. The raw scores range from 0 to 21. The longest score, the last item, has a maximum of 7. We calculated three outcomes from this test: (i) LNS pre-task: Total number of correctly recalled attempts pre-task; (ii) LNS post-task: Total number of correctly recalled attempts post-task; and (iii) Retention Rate: percentage of the score after the TSST/control task compared to the total score before the task (WM post-task/WM pre-task × 100).

### Biochemical Analyses

Salivary samples were collected using salivettes (Sarstedt, Nümbrecht, Germany). Participants were instructed to keep the cotton swab in their mouths for exactly 2 min, not chew the cotton, and move the swab around in a circular pattern to collect saliva from all the salivary glands. The samples were kept in the refrigerator until they were centrifuged at 3,000 rpm for 5 min, resulting in a clear supernatant of low viscosity that was stored at −80 C until the analyses. For each subject, all C and sAA samples were analyzed in the same trial. C levels were analyzed using the commercial enzymeimmunoassay kit from Salimetrics (Newmarket, United Kingdom). The sensitivity of the assay was <0.007 μg/dL, and the intra- and inter-assay coefficients of variation were all below 10%. C levels were expressed in nmol/L.

The sAA concentration was measured by using an enzyme kinetic method with the commercial salivary sAA assay kit from Salimetrics (United States). Assay sensitivity was 0.4 U/mL. Inter- and intra-assay variation coefficients were all below 10%. sAA concentrations were expressed in U/mL.

Two capillary blood samples taken at habituation (−20 min) and recovery period (+55) were employed to measure glucose concentration using a glucose monitoring system (Onetouch ultraeasy life Scan Europe 6300 Zug. Switzerland. AW 0639870).

### Statistical Analyses and Data Management

Student’s *t*-test was used to analyze socio-demographic differences between stress and control participants, whereas differences in educational level and marital status were analyzed with Chi-square tests.

To investigate the effects of stress on self-efficacy and situational appraisal, multivariate analyses of variance (MANOVA) were performed with each self-efficacy (i.e., capacity, self-confidence, and motivation) and situational appraisal (i.e., effort required, frustration, own performance, stress perceived, difficulty, importance of doing the task, and expected outcome) item as dependent variable and Condition as independent variable.

In order to investigate the effects of stress on mood, glucose levels, and WM performance (LNS), ANOVAs for repeated measures were performed, with Condition as a between-subject factor and Time (pre vs. post) as a within-subject factor.

Because C and sAA values did not show a normal distribution, they were log transformed. ANOVAs for repeated measures with Condition (stress vs. control) as a within-subject factor and Time (−15, +5, +15, +25, +35, +45, +50 min) as a between-subject factor were performed to assess the C and sAA levels. Partial eta squared (η^2^) is reported as a measure of effect sizes for ANOVAs (Cohen, 1973).

Finally, to assess whether the psychobiology response to the stressor was related to WM performance, two regression analyses were performed only for the participants in the stress condition. In one regression we included in Step 1 C_*reactivity*_ (maximum C levels after the stressful task minus baseline period) and sAA_*reactivity*_ (maximum value on the stressful task minus baseline period) and in Step 2 the interaction between C_*reactivity*_ and sAA_*reactivity*_. In the second regression, we included in Step 1 the positive affect reactivity (PAreactivity) and negative affect reactivity (NAreactivity), that is, the positive or negative affect post task minus positive or negative affect pre task, respectively.

Two outliers in the mood data, two outliers in the glucose data, one outlier in the cortisol data, and two outliers in the sAA data were excluded from the analyses because their values differed by more than 3 *SD*. from the mean. In addition, one woman was removed because her scores on the WM retention rate differed by more than 3 *SD*.

Greenhouse–Geisser was used when the requirement of sphericity was violated in the ANOVA for repeated measures. *Post hoc* planned comparisons were performed using Bonferroni adjustments for the *p*-values. Analyses were carried out using SPSS 25.0. All *p* values reported are two-tailed. The level of significance was fixed as <0.05. For easy interpretation, the values in the figures represent raw values.

## Results

### Preliminary Analyses

No condition effects were found on age (*p* = 0.235), education level (*p* = 0.356), SES (*p* = 0.594), BMI (*p* = 0.643), marital status (*p* = 0.353), or years of diagnosis (*p* = 0.797).

#### Self-Efficacy

The MANOVA showed significant differences between conditions in capacity [*F*_(__1_, _47__)_ = 10.144, *p* = 0.003, η^2^*_*p*_* = 0.178] and self-confidence [*F*_(__1_, _47__)_ = 4.215, *p* = 0.046, η^2^*_*p*_* = 0.082]; participants in the stress condition reported less ability and less confidence about performing the task successfully than those in the control condition. No significant differences in motivation [*F*_(__1_, _47__)_ = 0.748, *p* = 0.391, η^2^*_*p*_* = 0.016] were observed.

#### Situational Appraisal

The MANOVA showed that the stress task was perceived as more stressful [*F*_(__1_, _44__)_ = 7.796, *p* = 0.008, η^2^*_*p*_* = 0.151], frustrating [*F*_(__1_, _44__)_ = 17.438, *p* < 0.001, η^2^*_*p*_* = 0.284], difficult [*F*_(__1_, _44__)_ = 65.838, *p* < 0.001, η^2^*_*p*_* = 0.599], and requiring more effort [*F*_(__1_, _44__)_ = 20.777, *p* < 0.001, η^2^*_*p*_* = 0.321] than the control task. In contrast, participants in the control condition thought they performed the task better [*F*_(__1_, _44__)_ = 36.529, *p* < 0.001, η^2^*_*p*_* = 0.454] and had better results [*F*_(__1_, _44__)_ = 35.675, *p* < 0.001, η^2^*_*p*_* = 0.448] than those in the stress condition. No significant differences in the importance of doing the task were observed between conditions [*F*_(__1_, _44__)_ = 0.390, *p* = 0.536, η^2^*_*p*_* = 0.009].

#### Glucose Levels

Regarding the glucose levels, the ANOVA for repeated measures showed a main effect of Time [*F*_(__1_, _46__)_ = 5.857, *p* = 0.020, η^2^*_*p*_* = 0.113]. However, no effects of Condition [*F*_(__1_, _46__)_ = 0.202, *p* = 0.655, η^2^*_*p*_* = 0.004] or the Time × Condition interaction [*F*_(__1_, _46__)_ = 0.537, *p* = 0.468, η^2^*_*p*_* = 0.012] were found. In both conditions, participants showed significantly lower glucose levels after the task than before it (Pre task: *M* = 153.36, *SEM* = 6.86; Post task: *M* = 142.76, *SEM* = 7.52).

### Psychobiological Stress Response

#### Positive and Negative Affect

The ANOVA for repeated measures did not show effects of Condition [*F*_(__1_, _46__)_ = 0.116, *p* = 0.735, η^2^*_*p*_* = 0.003] on positive affect (PA). Although, the Time factor failed to reach significance [*F*_(__1_, _46__)_ = 3.972, *p* = 0.052, η^2^*_*p*_* = 0.079], the Time × Condition interaction was significant [*F*_(__1_, _46__)_ = 6.447, *p* = 0.015, η^2^*_*p*_* = 0.123]. *Post hoc* analyses showed that, overall, participants presented less positive affect after the stress task than before it. No differences were found between conditions before and after the task. However, participants in the stress condition reported significantly less PA after the stress task than before it (*p* = 0.003). No differences were observed in the control condition (*p* = 0.695) (Table 2).

**TABLE 2:** Mood changes (mean ± SEM) for total sample and both conditions.

	Time	Total sample	Stress condition	Control condition	*p*
Positive affect	Pre stress	31.195 (0.977)	31.870 (1.410)	30.520 (1.353)	0.493
	Post stress	29.589 (1.210)	28.217 (1.746)	30.960 (1.675)	0.263
Negative affect	Pre stress	16.631 (0.440)	16.636 (0.635)	16.625 (0.608)	0.990
	Post stress	17.634 (0.694)	19.227 (1.002)	16.042 (0.960)	0.027

Regarding negative affect (NA), there were no significant effects of Time [*F*_(__1_, _44__)_ = 2.033, *p* = 0.161, η^2^*_*p*_* = 0.44] or Condition [*F*_(__1_, _44__)_ = 2.911, *p* = 0.091, η^2^*_*p*_* = 0.064], but the Time x Condition interaction was significant [*F*_(__1_, _44__)_ = 5.083, *p* = 0.029, η^2^*_*p*_* = 0.104]. *Post hoc* analysis showed no differences between conditions before the task. After the task, participants in the stress condition showed higher NA than participants in the control condition. Moreover, participants in the stress condition reported higher NA after the stress task than before it (*p* = 0.014). No differences were observed in the control condition (*p* = 0.552).

#### Cortisol

**FIGURE 2:**
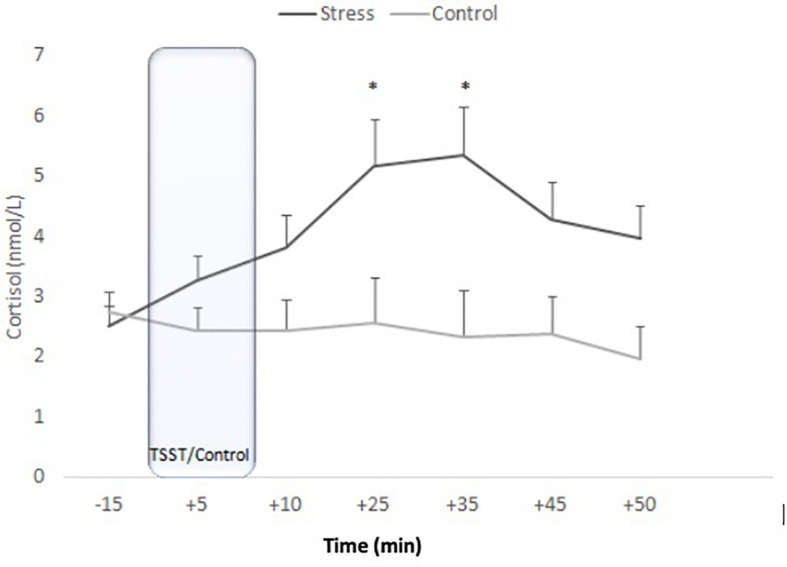
Means of salivary corliaol concentrations for stress and control conditions. Error bars represent standard error of means (^∗^*p* < 0.05).

The ANOVA for repeated measures did not show an effect of Condition [*F*_(__1_, _43__)_ = 2.850, *p* = 0.099, η^2^*_*p*_* = 0.062], although Time [*F*_(__2_._101_, _90_._328__)_ = 3.167, *p* = 0.044, η^2^*_*p*_* = 0.069] and the Time x Condition interaction were significant [*F*_(__2_._101_, _90_._328__)_ = 6.807, *p* = 0.001, η^2^*_*p*_* = 0.137]. *Post hoc* analyses showed that C levels were significantly higher in the stress condition than in the control condition +25 (*p* = 0.026), +35 (*p* = 0.019), and +50 (*p* = 0.048) min after the onset of the task. In addition, in the stress condition, there were no significant differences between C levels before and during the stress task (−15 vs. +5 samples: *p* = 0.147). However, C levels increased significantly from +10 min to +25 min (*p* = 0.003). After peaking at +35, cortisol levels started to decrease (+35 vs. +50 samples: *p* = 0.032) until finally reaching baseline levels (+ 50 vs. −15 samples: *p* > 0.99). In the control condition, no significant differences were observed (all *p* > 0.147) (Figure 2).

#### sAA

**FIGURE 3:**
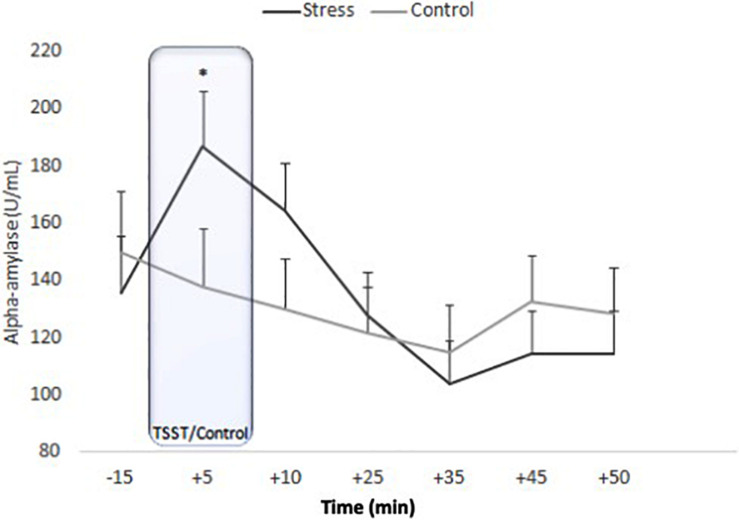
Means of salivary alpha-amylase concentrations for stress and control conditions. Error bars represent standard error of means (^∗^*p* = 0.002).

ANOVA for repeated measures showed an effect of Time [*F*_(__4_._379_, _170_._765__)_ = 11.900, *p* < 0.001, η^2^*_*p*_* = 0.234] and the Time x Condition interaction [*F*_(__4_._379_, _170_._765__)_ = 5.171, *p* < 0.001, η^2^*_*p*_* = 0.117]. No effects of Condition were observed [*F*_(__1_, _39__)_ = 0.069, *p* = 0.795, η^2^*_*p*_* = 0.002]. In the stress group, sAA levels started to increase at −15 min and were significantly higher at +5 min (*p* = 0.002). After peaking at +5 min, sAA levels significantly decreased (+5 vs. +35 samples: *p* < 0.001) and then remained stable (+35 vs. +50 samples; *p* > 0.99). In the control condition, no significant differences were observed (all *p* > 0.137) (Figure 3).

### Working Memory Performance

**FIGURE 4:**
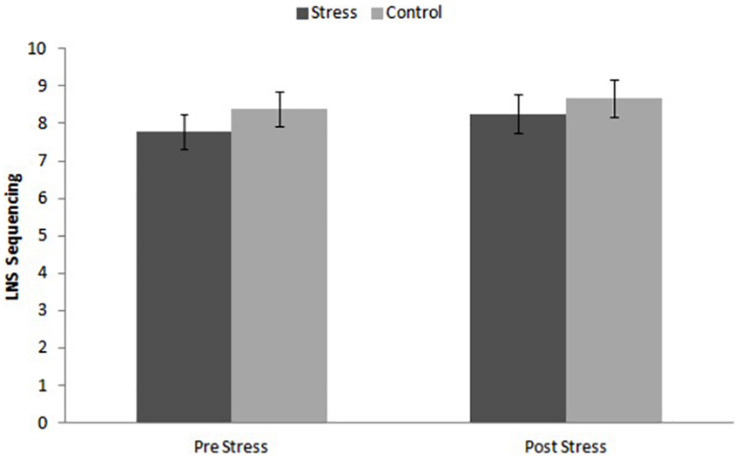
Performance on Letter-Number sequencing (LNS) tor stress and control conditions before and after the task.

The ANOVA for repeated measures did not show significant effects of Time [*F*_(__1_, _46__)_ = 1.946, *p* = 0.170, η^2^*_*p*_* = 0.041], Condition [*F*_(__1_, _46__)_ = 0.657, *p* = 0.422, η^2^*_*p*_* = 0.014], or the Time × Condition interaction [*F*_(__1_, _46__)_ = 0.133, *p* = 0.717, η^2^*_*p*_* = 0.003]. The performance in both groups was similar (Figure 4).

### Relationship Between the Psychobiological Response and WM Performance

In the stress condition, the WM retention rate was not related to PA_*reactivity*_ (B = −0.367; *p* = 0.108), NA_*reactivity*_ (B = 0.194; *p* = 0.383), C_*reactivity*_ (*B* = 0.055, *p* = 0.831), or sAA_*reactivity*_ (*B* = 0.038, *p* = 0.882). However, the interaction term C_*reactivity*_ × sAA_*reactivity*_ was negatively associated with the WM retention rate (*B* = −0.549, *p* = 0.026). Thus, participants in the stress condition who showed higher C and sAA responses to the TSST had lower WM performance.

## Discussion

The main purpose of the present study was to characterize the response of older men and women with T2D to an acute psychosocial stressor (i.e., TSST) and its effects on WM performance. As we expected, the TSST provoked significant mood changes and C and sAA responses in participants who were exposed to it, compared to those in the control condition. In addition, although stress did not affect WM performance, the WM retention rate was negatively related to the interaction between the C and sAA responses to stress.

Our results confirm that a psychosocial stressor (TSST) was able to produce a “typical” or characteristic psychobiological response in medically treated T2D patients. At the subjective level, the TSST was perceived as more stressful, frustrating, difficult, and requiring more effort than the control task, and the participants in the stress condition perceived that they had less ability to perform the task successfully. However, both stress and control participants showed a similar motivation to perform the task because they gave the same importance to their performance. Furthermore, the TSST elicited mood changes characterized by decreases in positive mood and increases in negative mood. This pattern confirms that this laboratory psychosocial stressor is able to generate a similar mood response in T2D patients to the one reported for healthy older adults (Hidalgo et al., 2020).

The TSST also provoked a clear C response in T2D patients. This result is consistent with a previous study (Faulenbach et al., 2011) reporting that, although C did not increase right after the stress task, the increase was detectable 30 min after the TSST in T2D patients. It is worth mentioning that in Faulenbach et al.’s study, T2D patients were subjected to the stress and control conditions in two different states (fasting or postprandial state). C increases were found in both states, although they were slightly greater in the fasting state. In our study, participants were in a short fasting state (1–2 h fasting period), and they showed significant reductions in their glucose levels throughout the session, regardless of the condition. Faulenbach et al. (2011) reported stable glucose concentrations in the TSST condition and slight reductions in the control condition in the fasting state (a 10 h overnight fasting period, including stopping medication); in the postprandial state, they found higher glucose levels in the stress session than in the control session. In our case, after exposure to the TSST, participants in both conditions showed glucose decreases (4.52%) fairly similar to those in non-T2D groups (5.6%) assessed in our lab in other studies (unpublished data). It is worth noting that participants in this study were periodically supervised by their doctors and maintained adequate metabolic control.

In addition, in our study, participants showed a sAA response to the TSST. This finding is consistent with results found in healthy older people (Almela et al., 2011; Pulopulos et al., 2015). However, Strahler et al. (2010) found an attenuated sAA response to TSST in this age group. To the best of our knowledge, the current study is the first one to investigate the sAA response to an acute psychosocial stressor in older people with T2D. As in healthy older people, our results support the idea that the TSST also provokes a greater sympathetic-adrenal-medullary system response than the control task in older T2D subjects.

Until now, only a few studies have been carried out on sAA, with heterogenous results in terms of differences between T2D and healthy people (Panchbhai et al., 2010; Shankaraiah and Reddy, 2011). We found higher levels of sAA in older adults with T2D undergoing acute psychosocial stress, compared to older subjects with T2D in a control condition. Our results are in line with those described in healthy older people (Pulopulos et al., 2015) and extend to T2D patients. In addition, they provide new information about the acute sAA response in T2D patients, and they confirm the relationship previously reported between sAA and perceived stress assessed by the Perceived Stress Scale (Siddiqui et al., 2015).

We did not find a significant effect of the TSST on WM performance or a relationship between the C response and WM performance. Our findings agree with other studies on both C administration (Wolf et al., 2001; Porter et al., 2002; Yehuda et al., 2007) and a stress-induced C response (Pulopulos et al., 2015) in healthy older people. Despite this, these results contrast with the conclusions observed in most studies with young male populations exposed to the TSST (Luethi et al., 2009; Engert et al., 2011) and the cold-pressor test (Schoofs et al., 2009; Becker and Rohleder, 2019). The lack of stress effects on WM performance in our sample of older people with T2D extends previous findings found in healthy older people and supports the idea that older people may be less sensitive to the effects of stress on memory than young people, as we previously reported for WM (Pulopulos et al., 2015) and declarative memory (Pulopulos et al., 2013; Hidalgo et al., 2014, 2015) performance. This lack of stress effects on WM performance could be due to an age-related dysregulation of HPA-axis activity (Mizoguchi et al., 2009) and functional changes in the amygdala and hippocampus (Mather, 2006; Murty et al., 2009; St. Jaques et al., 2009) in older people. However, this lack of acute stress effects on WM in older people with T2D should be interpreted with caution, given that a healthy control older group was not included in the present study.

Although we failed to find a stress effect on WM performance, the interaction term between the C and sAA reactivities to the stressor was negatively associated with WM performance. Thus, participants who responded to stress with higher cortisol and sAA levels had worse WM performance. It has been well established that WM depends on prefrontal cortex functioning (Galloway et al., 2008), and that this brain area is affected by the glucocorticoid action and noradrenergic activation in response to stress (Patel et al., 2000; Schoofs et al., 2008). Hence, it is conceivable that this type of memory would be affected by the activation of both stress systems; therefore, the WM performance could be related not only to the HPA-axis or SNS action separately, but also to the interaction between these two systems. To the best of our knowledge, this association has not been previously reported in patients with T2D. Future studies are needed to support this association.

The present study has some limitations. To avoid as many confounding factors as possible, we were conservative and selected a homogeneous T2D sample in terms of age, sex, and other relevant sociodemographic characteristics. Consequently, the number of participants included was limited, which meant that we could not study possible gender differences in the acute stress effects on WM in older people with T2D. Therefore, future studies should include bigger sample sizes and study the role of gender in this clinical population. In addition, although this study has a control group with T2D patients who did not undergo stress, further studies would benefit by also including other control group with healthy people. In spite of these limitations, the current study makes it possible to advance the knowledge about the characteristics of the acute response to psychosocial stress in medically treated T2D. In fact, our results suggest a psychobiological response similar to the one found in healthy older people, and different from that of young people. This similitude extends to the stress effects on WM for this age group. WM has great relevance in older people, and particularly in the T2D population, because many therapeutic actions are based on information that is necessary for a high degree of patient self-management. In sum, this study provides interesting findings about the psychobiological response to acute psychosocial stress in older T2D men and women. The results provide empirical evidence about the cognitive and physiological response in older people with T2D with adequate medical supervision.

## Data Availability Statement

The raw data supporting the conclusions of this article will be made available by the authors, without undue reservation, to any qualified researcher.

## Ethics Statement

The studies involving human participants were reviewed and approved by the La Fe- Hospital Clínico Universitario- Instituto de Investigación Universitario- Universitat de València. The patients/participants provided their written informed consent to participate in this study.

## Author Contributions

AS, VH, and SP-P: conceptualization and design. SP-P and TM: recruitment and data collection. LV and MZ-F: data curation and writing—original draft preparation. AS and VH: writing—review and editing. AS and JN: supervision. AS: project administration and funding acquisition. All authors have read and agreed to the published version of the manuscript.

## Conflict of Interest

The authors declare that the research was conducted in the absence of any commercial or financial relationships that could be construed as a potential conflict of interest.
